# Peripheral neuropathy in a case with CADASIL: a case report

**DOI:** 10.1186/s12883-018-1131-3

**Published:** 2018-08-31

**Authors:** Yusuke Sakiyama, Eiji Matsuura, Yoshimitsu Maki, Akiko Yoshimura, Masahiro Ando, Miwa Nomura, Kazuya Shinohara, Ryuji Saigo, Tomonori Nakamura, Akihiro Hashiguchi, Hiroshi Takashima

**Affiliations:** Department of Neurology and Geriatrics, Kagoshima University Graduate School of Medical and Dental Sciences, 8-35-1 Sakuragaoka, Kagoshima City, Kagoshima 890-8520 Japan

**Keywords:** CADASIL, Peripheral neuropathy, Multiple mononeuropathy, *NOTCH3*, Granular osmiophilic material (GOM)

## Abstract

**Background:**

Cerebral autosomal dominant arteriopathy with subcortical infarcts and leukoencephalopathy (CADASIL) is characterized clinically by central nervous system dysfunctions. It is unclear whether CADASIL is involved in peripheral neuropathy.

**Case presentation:**

A 67-year-old Japanese man with stepwise progression of sensory and motor neuropathy was admitted to our hospital. Peripheral neuropathy of the extremities was detected through electrophysiological and pathological studies, and brain magnetic resonance imaging revealed bilateral periventricular ischemic and thalamic hemorrhagic lesions. We diagnosed CADASIL after detecting granular osmiophilic material in the walls of the endoneurial vessels morphologically and identifying a heterozygous *NOTCH3* mutation p.Arg75Pro.

**Conclusions:**

CADASIL is to be included in the work-up of not classified peripheral neuropathies.

**Electronic supplementary material:**

The online version of this article (10.1186/s12883-018-1131-3) contains supplementary material, which is available to authorized users.

## Background

Cerebral autosomal dominant arteriopathy with subcortical infarcts and leukoencephalopathy (CADASIL) is an autosomal dominant disorder. This disease caused by mutations in the Notch3 gene that encodes a transmembrane protein expressed in vascular smooth muscle cells [1]. CADASIL patients have various clinical symptoms of central nerve systems, such as migraine, recurrent stroke, progressive cognitive decline, and psychiatric disturbance. Because it is unclear whether CADASIL is involved in peripheral neuropathy, we expand the phenotype of CADASIL by reporting electrophysiological and pathological features of CADASIL patient with peripheral neuropathy.

## Case presentation

**Fig. 1:**
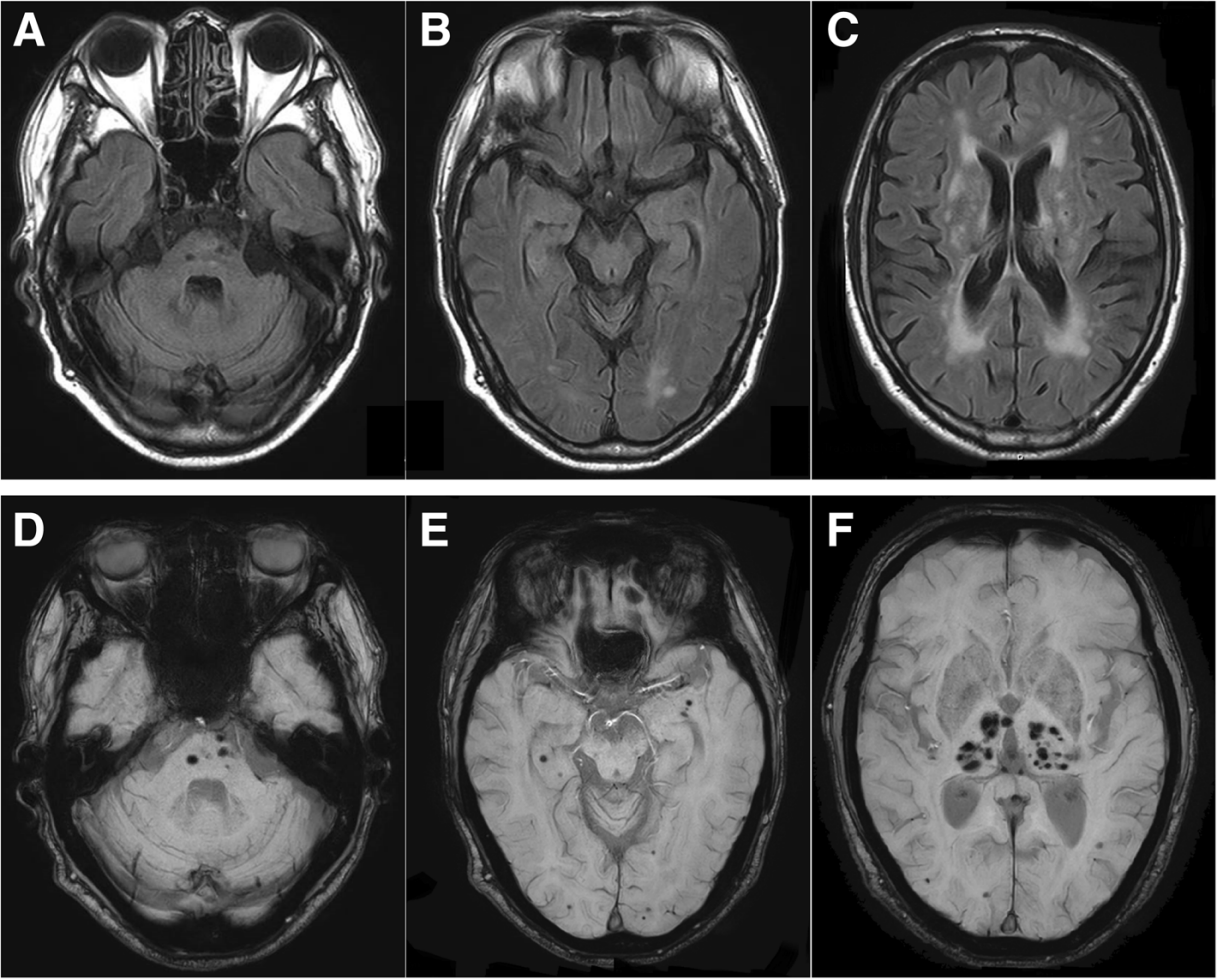
Brain-MRI findings of our patient. **a**–**c** Axial FLAIR image showing the bilateral ischemic lesions in pons, periventricular white matter and deep white matter. **d**–**f**: Axial susceptibility-weighted imaging (SWI) showing multiple micro-bleeding lesions in pons, bilateral subcortical white matter and bilateral thalamus

A 67-year-old man had the first episode of left leg weakness at the age of 65 years and subsequently experienced numbness of the bilateral fourth and fifth fingers (Additional file 1). At the age of 66, he developed right leg weakness and difficulty in walking and was admitted to our hospital. Neurological examination revealed no abnormalities in orientation, memory, or cranial nerves. Mild weakness was noted in the distal muscle of the upper limbs, and pronator drift test was positive in the left upper limb. Lower limb muscle weakness was moderate and particularly noticeable in the left anterior tibialis muscle. Deep tendon reflexes were decreased at the triceps and brachioradialis and absent at the knees and ankles. Babinski sign was positive. Pes cavus and toe clawing were absent. There were no finger tremors. A sensory examination showed bilateral hypoesthesia in the lower leg region, below the knees and bilateral numbness of the fourth and fifth fingers. No abnormalities in the urinary tract were found. The ankle-brachial index of blood pressure was normal. The results of a blood study revealed a mild inflammatory reaction and elevated serum proteinase-3-anti-neutrophil cytoplasmic antibody (PR3-ANCA; 4.0 U/mL; normal range, < 3.5 U/mL). Complete blood count and CRP level were normal. His liver and renal function were normal. Blood sugar was 97 mg/dl and HbA1c was 6.5%. A nerve conduction study was performed at a skin temperature of approximately 30 °C (Table 1). Distal motor and sensory nerve palm latency (DL) were prolonged in the left median nerve, and a slight decrease in sensory conduction velocity (SCV) was noted. Compound muscle action potentials (CMAP) at stimulation points above the elbow and sensory nerve action potentials (SNAP) were undetectable in the left ulnar nerve. These electrophysiological abnormalities were consistent with multiple mononeuropathy. Brain magnetic resonance imaging showed asymptomatic multiple cerebral infarctions and multiple micro-bleeding lesions in the white matter (Fig. 1). His symptoms did not improve after methylprednisolone pulse therapy and oral prednisolone (30 mg/day) and azathioprine (50 mg/day) therapies.

**Table 1:** Summary of electrophysiological data on first admission

		Left median	Left ulnar	Left tibial
DL (ms)		*4.7*	3.9	3.7
CMAP (mV) dis./prox.		6.1/5.4	4.5/*N.D.*	12.6/8.8
MCV (m/s)		53.0		43.4
F-latency (ms)		28.4	*29.7*	51.8
F-frequency (%)		100	68	100
	Left median	Left ulnar	Left sural	Right ulnar
SNAP (μV)	9.3	*N.D.*	6.9	*N.D*.
SCV (m/s)	44.2		43.9	

*DL* distal latency, *CMAP* compound muscle action potential, *MCV* motor conduction velocity, *SNAP* sensory nerve action potential, *SCV* sensory conduction velocity. *N.D.* not detected

**Fig. 2:**
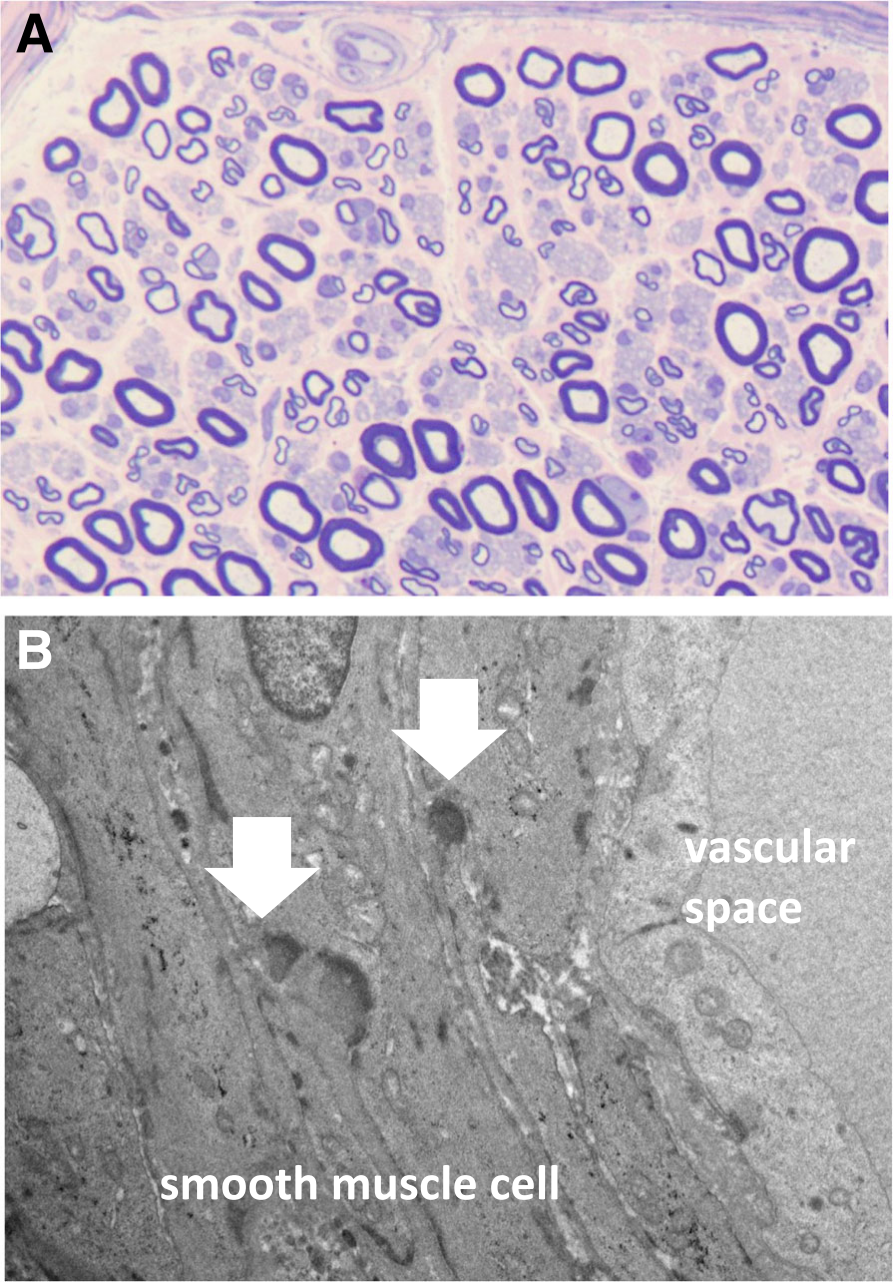
Pathological investigations on right sural nerve biopsy. The density of myelinated fibers slightly reduced (7303/mm^2^; normal range 7500–11,000 fibers/mm^2^), mostly damaged those of large diameter. **a**: Toluidine blue-stained semithin epon section. Some axon with thickest myelin and myelin ovoids appeared, suggesting axonal degeneration. Demyelinated fiber was absent. There were no inflammatory cell and subperineurial edema. **b**: Electron microscope study. Granular osmiophilic material (arrows) was viewed in the electron microscope, deposited between the smooth muscle cells surrounding a small artery in epineurial connective tissue

The pathological findings of a right sural nerve showed neither inflammatory cells around vessels nor any rupture of the inner elastic plate. The loss of smaller fibers and thickening of the blood vessel wall were not observed. Electron microscopy showed granular osmiophilic material (GOM) in the wall of endoneurial small vessels, which indicated peripheral neuropathy resulting from CADASIL (Fig. 2). The heterozygous missense mutation p.Arg75Pro was detected in exon 3 of the *NOTCH3* gene in the leukocyte DNA of the patient. No family history displayed cerebrovascular diseases or peripheral neuropathies. His father was diagnosed as heart failure of unknown etiology.

There was no history of smoking or drinking.

## Discussion and conclusion

We successfully diagnosed CADASIL in a Japanese case with peripheral neuropathy and the p.Arg75Pro mutation of the *NOTCH3* gene. The pathogenic mutation was previously reported as a cause of CADASIL in Japanese and Korean patients [2, 3] and most frequently found in Japanese patients with CADASIL. None of Japanese cases had peripheral neuropathy [4]. The pathophysiological mechanisms of brain lesions in CADASIL remain unclear. GOM accumulation in the small arterioles of the brain is a pathological feature of CADASIL [1]. Notch3 is predominantly expressed in vascular smooth muscle cells in adults [1, 5]. Previous immunoelectron microscopic examination revealed aggregated Notch3 protein in proximity to GOM deposits; however, it is unclear whether Notch3 protein is included in GOM [6–8]. While peripheral neuropathy is usually not seen in the patients with CADASIL, a few previous studies reported the association between CADASIL and peripheral neuropathy [5]. The study suggested axonal damage in peripheral nerve with clinical and electrophysiological findings. Our electron microscopic study revealed the deposition GOM in the wall of endoneurial small vessels in the peripheral nerve. Electrophysiological study showed multiple mononeuropathy, suggesting vascular dysfunction, while there was no inflammatory cell infiltration in the biopsied nerve. Novel protein–protein interactions caused by *NOTCH3* mutations has been suggested in vascular function. The dysfunction of blood vessels may induce hemodynamic abnormalities with low-grade chronic ischemia in peripheral nerves [5, 9]. We excluded other causes of chronic progressive axonal neuropathy, including of diabetes mellitus neuropathy, alcoholic neuropathy, ANCA–associated vasculitis, and drug or toxin.

To investigate the frequency of CADASIL in the patients with peripheral neuropathy, we performed genetic analysis of 434 patients of clinically suspected hereditary neuropathy by whole exome sequencing. However, *NOTCH3* pathogenic mutation was not found among the patients. In the previous study of CADASIL cases with peripheral neuropathy, subcortical infarcts and/or leukoencephalopathy were detected by brain MRI [5]. In other words, CADASIL cases presenting just with peripheral neuropathy have abnormal lesions in brain MRI. Although we didn’t detect *NOTCH3* mutation in the patients with hereditary neuropathy, the patients with neuropathy and brain lesions have better to be analyzed for the *NOTCH3* mutation.

In conclusion, CADASIL is to be included in the work-up of not classified peripheral neuropathies. Brain MRI and *NOTCH3* gene analysis may provide a definite diagnosis for these neuropathies.

## Additional file

Additional file 1: Time line. (DOCX 12 kb)

## Abbreviations

CADASIL: Cerebral autosomal dominant arteriopathy with subcortical infarcts and leukoencephalopathy
CMAP: Compound muscle action potentials
CRP: C-reactive protein
DL: Distal latency
GOM: Granular osmiophilic material
PR3-ANCA: Proteinase-3-anti-neutrophil cytoplasmic antibodies
SCV: Sensory conduction velocity
SNAP: Sensory nerve action potentials
