# Supplementary material for: Peripheral neuropathy in a case with CADASIL: a case report
Source: BMC Neurol. 2018 Aug 31;18:134. doi: 10.1186/s12883-018-1131-3 (PMC6117928; doi:10.1186/s12883-018-1131-3)
Supplement: Supplementary file 1 — Time line. (DOCX 12 kb) [file 12883_2018_1131_MOESM1_ESM.docx]

Time Line

| Date | Event | Test | Interventions |
| --- | --- | --- | --- |
| 10/19/2015 | First visit to Kagoshima University Hospital |  |  |
| 10/16/2015 |  | Brain MRI |  |
| 1/4/2016 | Admission date |  |  |
| 1/5/2016 |  | Nerve Biopsy date |  |
| 1/20/2016 |  |  | Steroid treatment |
| 1/26/2016 | Discharge |  |  |
